# Protein energy-wasting associated with nephrotic syndrome – the comparison of metabolic pattern in severe nephrosis to different stages of chronic kidney disease

**DOI:** 10.1186/s12882-020-02003-4

**Published:** 2020-08-14

**Authors:** Anna Matyjek, Slawomir Literacki, Stanislaw Niemczyk, Aleksandra Rymarz

**Affiliations:** 1 Department of Internal Medicine, Nephrology and Dialysis, Military Institute of Medicine, Szaserów Street 128, 04-141 Warsaw, Poland; 2 Department of Laboratory Diagnostic, Military Institute of Medicine, Warsaw, Poland

**Keywords:** Nephrotic syndrome, Body composition, Bioimpedance spectroscopy, Protein-energy wasting, Hypercatabolism, Lean tissue, Hyperphosphatemia, Hyperuricemia

## Abstract

**Background:**

Nephrotic syndrome (NS) is associated with a hypercatabolic state expressed as an exacerbated degradation of muscle mass. However, the clinical significance of this phenomenon has not yet been investigated.

The aim of the study was to evaluate the nutritional status of patients with severe NS (defined as nephrotic range proteinuria with hypoalbuminemia ≤2.5 g/dL) and estimated glomerular filtration rate (eGFR) ≥45 mL/min/1.73 m^2^ in comparison to patients in different stages of chronic kidney disease (CKD).

**Methods:**

Twenty men with severe NS (NS group) and 40 men without proteinuria similar in term of serum creatinine (control group) were included into the study. A retrospective cohort of 40 men with CKD stage G4 (PreD group) and 20 haemodialysis men (HD group) were added to the analysis after matching for age, height and weight using propensity score matching. The bioimpedance spectroscopy and biochemical nutritional markers were evaluated.

**Results:**

Nephrotic patients had a significantly lower lean tissue mass (LTM; *p* = 0.035) and index (a quotient of LTM over height squared, LTI; *p* = 0.068), with an expected deficiency of LTM by 3.2 kg, and LTI by 0.9 kg/m^2^ when compared to the control group. A significant lean tissue deficit (defined as LTI below the lower limit of the reference range by 1.0 kg/m^2^) was observed in 12.5% of patients in the control group in comparison to 31.7% with advanced CKD (PreD+HD; *p* = 0.032) and 50% with NS (*p* = 0.003). NS group presented with higher phosphorus (*p* = 0.029), uric acid (*p* = 0.002) and blood urea (*p* = 0.049) than the control group. Blood urea was strongly negatively correlated with LTM in NS (*r* = − 0.64, *p* = 0.002). Nine nephrotic patients (45%) were identified as hypercatabolic based on severe hyperphosphatemia (> 5.0 mg/dL) and/or hyperuricemia (> 8.0 mg/dL), and were characterized by higher blood urea and lower prealbumin, as well as LTM lower by 5.6 kg than in less catabolic individuals.

**Conclusions:**

In term of lean tissue amount, NS group was more similar to advanced CKD than to the control group. We concluded that specific metabolic pattern with elevated phosphorus, uric acid and blood urea, and lean tissue deficiency may be defined as protein-energy wasting associated with nephrotic syndrome (neph-PEW).

## Background

Nephrotic syndrome (NS) is a rare clinical condition caused by glomerular filtration barrier damage due to glomerulopathy and is characterized by proteinuria exceeding 3.5 g/day, resulting in hypoalbuminemia, hyperlipidemia and oedema.

Pathophysiological studies have indicated that nephrotic syndrome is associated with a hypercatabolic state expressed as an exacerbated degradation of muscle mass, however, the clinical significance of this phenomenon has not yet been investigated [1, 2].

Pathophysiology of protein metabolism and its regulation in nephrotic syndrome has been studied in detail since the 1950s. It has been found that aside from urinary loss of albumin, an increased fraction of this protein is catabolized in renal tubular cells. The intensified hepatic synthesis is insufficient to counterbalance the overall deficit and the hypoalbuminemia is present until the proteinuria ameliorates [3–5]. Thus, it has been established that due to a loss of a large amount of protein, a decrease in the general body protein pool is observed in nephrotic patients [6]. As muscles are the main source of amino acids, necessary substrates for hepatic synthesis of lost plasma proteins, the catabolic state existing in nephrotic patients is manifested by a reduction in lean tissue [7]. However, the problem of hypercatabolism and malnutrition seems to be neglected in this population. This is probably due to the low incidence of glomerulopathies, estimated at 1–2 people per 100,000 (for primary glomerulopathies) with only about 50% of them displaying clinical signs of nephrotic syndrome and an even lower frequency with a severe course of the disease [8].

In contrast, in chronic kidney disease (CKD) the problem of malnutrition has been widely discussed for the last 20 years [9]. This has resulted in the establishment of a definition for protein-energy wasting syndrome (PEW) as a specific nutritional impairment accompanying CKD, by the Society of Renal Nutrition and Metabolism (ISRNM) in 2006. It has been recommended to follow anthropometric criteria (body mass index: BMI < 23 kg/m^2^, loss of body weight or fat, reduction of muscle mass), biochemical measurements (serum albumin < 3.8 g/dL, total cholesterol < 100 mg/dL, prealbumin < 30 mg/dL in dialysis subjects) and insufficient dietary energy intake [10].

The aim of the study was to evaluate the nutritional status of patients with severe nephrotic syndrome and normal or at most mildly-to-moderately impaired renal function in comparison to patients in different stages of CKD using biochemical measurements and the bioimpedance spectroscopy (BIS) technique. An additional purpose of the study was to define the metabolic pattern in severe NS and to determine simple markers of hypercatabolism in this group of patients.

## Methods

### Study population

Twenty adult men with severe nephrotic syndrome and eGFR ≥45 mL/min/1.73m^2^ (NS group), diagnosed for the first episode or relapse of NS after at least 3 years of complete remission and previous withdrawal of immunosuppressive agents, were included into the study. Severe nephrotic syndrome was defined as nephrotic range proteinuria (> 3.5 g/day) with serum albumin concentration ≤ 2.5 g/dL and oedema in physical examination. Nutritional status assessment was prior to dietary intervention, introduction of immunosuppressive agents and lipid-lowering drugs.

Control group consisted of 40 men without proteinuria and overhydration in physical examination, matched to nephrotic patients by age, weight, height and eGFR.

Exclusion criteria were severe cardiovascular, respiratory or liver disease, neoplasm, extreme obesity (BMI ≥40 kg/m^2^), special diet.

**Fig. 1:**
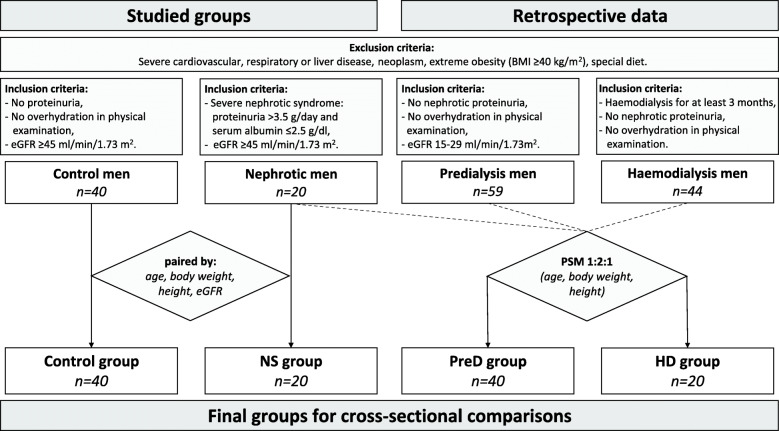
Flowchart of the study design. PSM (propensity score matching) – between nephrotic men (NS group) and retrospective data of patients with CKD stage G4 (predialysis men; 1:2 matching) or haemodialysis men (1:1 matching) resulted in the formation of PreD and HD group

The available data from the past study was used to form cohorts of patients with advanced CKD (stage G4-G5D). Accounting for exclusion criteria, cohorts of 59 predialysis men in CKD G4 stage and of 44 men haemodialyzed thrice-weekly for at least 3 months were selected. Subsequently, a propensity score matching technique (PSM 1:2 or 1:1) was used to match patients from these cohorts in terms of age, body weight and height to nephrotic patients to minimize the selection bias. The final groups analysed in the study were, as follows: NS group (20 men with severe nephrotic syndrome and eGFR ≥45 mL/min/1.73m^2^), control group (40 men without proteinuria with eGFR ≥45 mL/min/1.73m^2^), PreD group (40 non-nephrotic men in CKD stage G4, eGFR 15–29 mL/min/1.73m^2^) and HD group (20 men with end-stage renal disease undergoing maintenance haemodialysis therapy). A summary of the study design is presented in Fig. 1.

The study was conducted in accordance with the principles of the declaration of Helsinki. The study protocol was approved by the local bioethics committee. Written informed consent was signed by all study participants.

### Biochemical and body composition measurements

Serum albumin concentration (SA); total serum protein; prealbumin; blood urea; creatinine (in HD group before haemodialysis session); lipid profile including total cholesterol, triglycerides, low- (LDL) and high-density lipoproteins (HDL); uric acid (UA); phosphorus (Pi); and C-reactive protein were assessed and tested using the appropriate chemical analyser *(Cobas c501, Roche Diagnostic, Switzerland)*. Estimated glomerular filtration rate was calculated based on the Modification of Diet in Renal Disease (MDRD) formula.

Multifrequency bioimpedance spectroscopy examination was performed in all study participants. The BIS device (Body Composition Monitor, *Fresenius Medical Care, Germany*) was connected by skin electrodes placed on hand and foot on the same body side. In haemodialysis men with arteriovenous fistula, the side without vascular access was used to place the electrodes. A measurement was carried out on patients staying in a supine position for at least 5 min, for the HD group, before a mid-week haemodialysis session. The amount of lean tissue mass (LTM), lean tissue mass percentage (LTM%; a quotient of LTM and body weight, expressed as a percentage), lean tissue index (LTI; a quotient of lean tissue mass over height squared), lean tissue deficit (∆LTI; the difference between a patient’s LTI and the lower limit reference range for gender and age), adipose tissue mass (ATM), body cell mass (BCM), and overhydration (OH) were evaluated.

### Statistical analysis

Final study population was established using a PSM technique (nearest neighbour matching 1:1 or 1:2) for selection, with the closest similarities possible between patients with advanced CKD (predialysis and haemodialysis group) and the NS cohort in terms of age, body weight and height.

Continuous variables were described as mean and standard deviation (SD) if normally-distributed or median with interquartile range (IQR: 25–75% percentile) if distribution was non-normal. Categorical variables were presented as numbers (n) with percentages (%). Differences between four groups in terms of continuous variables were evaluated with analysis of variance (ANOVA) with Bonferroni post-hoc test (if normally-distributed data with homogeneity of variation) or Kruskal-Wallis analysis with post-hoc test (if non-normally distributed data or heterogenous variance despite the normal distribution). Chi-square (χ^2^) tests were used to compare categorical variables in either two (exact Fisher χ^2^ test) or four groups (Person χ^2^ test). The significant differences in qualitative data between the two groups were expressed as odds ratio (OR) with a 95% confidence interval (95% CI). The influence of confounding factors (age, BMI) on the BIS results was reduced by using an analysis of covariance (ANCOVA). Confounding variables were included in the analysis if *p*-value of a covariate was < 0.1. Linear relationships between two variables were estimated as Pearson’s correlation coefficient (r) or Spearman’s rank correlation coefficient (R), respectively. Significant hyperphosphatemia (hPi) was defined as serum phosphate concentration exceeding the mean value by three SD in the control group (Pi > 5.0 mg/dL), and hyperuricemia (hUA) as uric acid concentration of two SD above the mean value in the control group (UA > 8.0 mg/d). All tests were two-tailed, *p*-values < 0.05 indicated statistical significance.

Statistical analysis was performed using Statistica version 13.1. (*TIBCO Software Inc., USA*).

## Results

The study group included 20 men with severe nephrotic syndrome with mean proteinuria 11.5 ± 4.5 g/day and SA 1.9 ± 0.4 g/dL. The causes of severe NS in the group were, as follows: minimal change disease (MCD) in 10 patients (50%), membranous nephropathy (MN) in 6 patients (30%), AA amyloidosis in 2 patients (10%), focal segmental glomerulosclerosis (FSGS) in 1 patient (5%) and AL amyloidosis in 1 member of the NS group (5%).

Nephrotic men were an average age of 46 ± 17 years. In spite of the selection of similar patients, there was a significant difference between the groups in terms of age, and of borderline significance in terms of BMI. CKD was recognized in 5 nephrotic patients (secondary to glomerulopathy), and in 8 patients in the control group due to hypertension (*n* = 6) or nephrolithiasis (*n* = 2). A transient eGFR decrease – acute kidney injury (AKI) – was observed in an additional 2 patients in the NS group due to severe course of the disease. The delay between the onset of nephrotic syndrome and the nutritional assessment was less than 1 month in 50% of patients (short NS history), all of whom had MCD, and 1–3 months (long NS history) in the other 10 cases (Table 1). MCD patients (short history group) were significantly younger than the other nephrotic individuals (Additional file Table S1).

**Table 1:** Characteristics of the study population

Variable		NS *n = 20*	Control *n = 40*	PreD *n = 40*	HD *n = 20*	***p***-value
**History of NS:**						
**Short** (< 1 month)	n (%)	10 (50%)	–	–	–	–
**Long** (1–3 months)	n (%)	10 (50%)				
**Age** [years]	mean ± SD	46 ± 17	48 ± 18	59 ± 15	49 ± 16	**0.007**
	min-max	[22–79]	[19–86]	[19–83]	[19–78]	
**Body weight** [kg]	mean ± SD	83 ± 14	86 ± 18	86 ± 15	79 ± 16	0.413
	min-max	[60–105]	[53–126]	[53–115]	[56–106]	
**Height** [cm]	mean ± SD	175 ± 10	176 ± 8	172 ± 7	175 ± 5	0.197
	min-max	[155–187]	[158–194]	[154–192]	[167–185]	
**BMI** [kg/m^2^]	mean ± SD	27.2 ± 3.5	27.6 ± 4.9	29.1 ± 4.8	25.9 ± 5.2	0.084
	min-max	[21.5–35.0]	[18.1–38.1]	[19.3–38.3]	[18.1–35.8]	
**eGFR** [mL/min/1.73m^2^]	mean ± SD	83 ± 29	86 ± 24	22 ± 4	8 ± 3	**< 0.0001**
	min-max	[45–147]	[45–159]	[15–29]	[4–14]	
**Renal impairment:**						
**No**	n (%)	13 (65%)	32 (80%)			0.103^#^
**Yes, CKD**	n (%)	5 (25%)	8 (20%)	–	–	
**Yes, AKI**	n (%)	2 (10%)	0			
**Serum creatinine** [mg/dL]	median	1.1	1.0	3.1	7.4	< 0.0001*
	(IQR)	(0.9–1.4)	(0.9–1.2)	(2.7–3.7)	(5.5–11.3)	
	min-max	[0.6–1.7]	[0.6–1.7]	[2.4–4.7]	[4.5–14.2]	
**C-reactive protein** [mg/dL]	median	0.30	0.30	0.34	0.34	0.223*
	(IQR)	(0.10–0.80)	(0.10–0.34)	(0.10–0.60)	(0.30–0.80)	
	min-max	[0.01–1.90]	[0.01–2.10]	[0.10–4.90]	[0.10–2.17]	

The average values were presented as mean ± SD or median (IQR), as appropriate. Four groups were compared with ANOVA or Kruskal-Wallis test (*) for continuous variables, and chi-square test for categorical data (#). Statistically significant differences between groups (*p*-values < 0.05) were bolded

### PEW diagnosis according to ISRNM criteria

According to the ISRNM criteria based on SA, prealbumin, total cholesterol and BMI, the diagnosis of PEW was made more often in HD patients (45%), than in the control (20%, *p* = 0.046) and PreD (17.5%, *p* = 0.023) groups. All of the NS patients met, by definition, the SA criterion of PEW (Additional file Table S2).

### Laboratory measurements

**Fig. 2:**
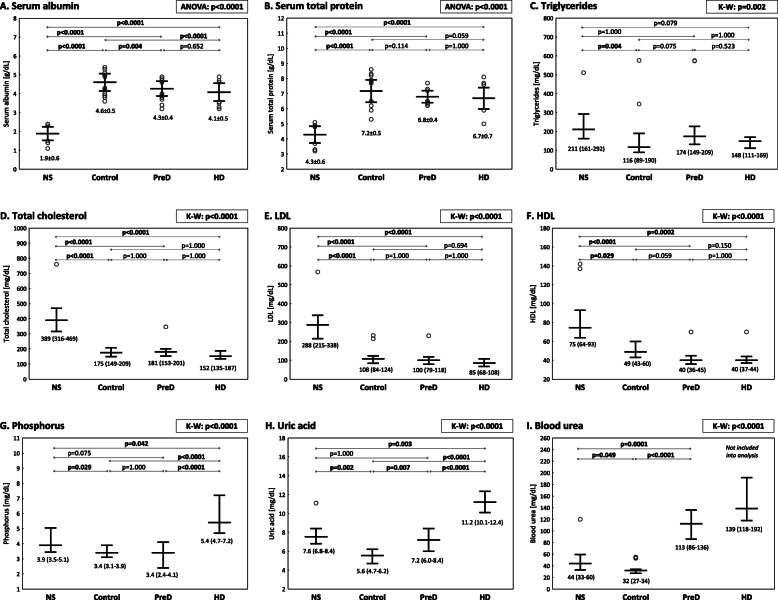
Comparison of biochemical nutritional and metabolic parameters between the groups (**a-i**). Serum albumin (**a**), serum total protein (**b**), triglycerides (**c**), total cholesterol (**d**), LDL (**e**), HDL (**f**), phosphorus (**g**), uric acid (**h**), blood urea (**i**). The average values of the relevant parameters were presented as mean with standard deviation or median with interquartile range, the outliers were marked as a circle. Comparisons of all groups were performed using analysis of variance (ANOVA) or Kruskal-Wallis test (K-W), respectively. The results of appropriate tests were presented as *p*-values. The differences between two selected groups in multiple pairwise comparisons (in Bonferroni post-hoc test or appropriate post-hoc test for K-W) were presented as *p*-values. *P*-values < 0.05 were bolded

Significant differences were found between the 4 groups in terms of serum albumin, total protein, lipids, phosphorus, uric acid and blood urea (Fig. 2a-i).

The most severe hypoalbuminemia and hypoproteinemia were seen in nephrotic patients in contrast to the other groups (*p* < 0.0001 for all pairwise comparisons). There was a lower albumin concentration observed in the PreD and HD groups in comparison to the control subjects (*p* = 0.004, and *p* < 0.0001, respectively). According to the PEW criteria, hypoalbuminemia was observed in 20% of HD in comparison to 2.5% of control patients (*p* = 0.038; OR = 9.75, 95% CI: 1.01–94.11). There was no significant difference in the incidence of hypoalbuminemia between the PreD and control group (10% vs 2.5%, *p* = 0.179) (Additional file Table S2). The difference between total serum protein in the control group and in PreD patients was close to statistical significance (*p* = 0.059).

Severe nephrotic syndrome was characterized by higher total cholesterol, LDL and HDL concentrations when compared to the other groups (*p* < 0.0001 for all pairwise comparisons). Nephrotic patients with short history of NS (and underlying MCD) had higher HDL levels than those with long history and other underlying nephropathies (Additional file Table S1). Triglycerides level was lower in the control group than in NS (*p* = 0.004). The differences in triglycerides concentrations between NS and HD group, as well as between the control and PreD group were close to statistical significance (*p* = 0.075, and *p* = 0.079, respectively). Total cholesterol levels below 100 mg/dL (indicating PEW) were not noted in any group (Additional file Table S2).

The highest phosphate and uric acid serum concentrations were observed in the HD group. There were also significantly higher Pi and UA levels in NS patients than in the control group (*p* = 0.029, and *p* = 0.002, respectively). A comparison of the NS and PreD groups revealed lower phosphorus concentration in advanced CKD – the difference was on the border of statistical significance (*p* = 0.075). Significant hyperphosphatemia (hPi, Pi> 5.0 mg/dL) and/or hyperuricemia (hUA, uric acid > 8.0 mg/dL) were not observed in any members of the control group, though were seen in 45% of patients with severe NS (9 patients), 52.5% of the subjects in the PreD group (21 patients) and in 100% of haemodialysis men (20 patients).

It was found that blood urea concentration was significantly higher in the NS group than in control subjects (*p* = 0.049), but lower than in the PreD group (*p* < 0.0001).

Apart from higher HDL and uric acid levels, patients with short NS history did not differ from those with longer duration of NS in term of laboratory measures of nutrition (Additional file Table S1).

### Body composition

**Fig. 3:**
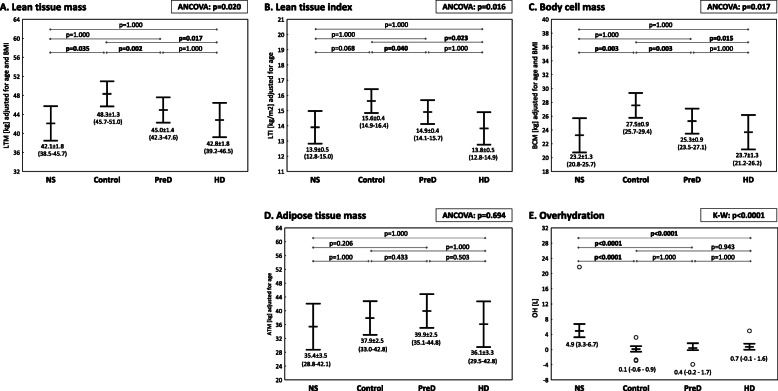
Comparison of bioimpedance spectroscopy parameters in the groups. Lean tissue mass adjusted to age and BMI (**a**), lean tissue index adjusted to age (**b**) body cell mass adjusted to age and BMI (**c**), adipose tissue mass adjusted to age (**d**), overhydration (**e**). Average values were presented as estimated marginal means with a 95% confidence interval for variables analysed with analysis of covariance (ANCOVA) or median with interquartile range if Kruskal-Wallis test (K-W) was used. Comparisons of all groups were presented as *p*-value. The differences between two selected groups in multiple pairwise comparisons (in Bonferroni post-hoc test or appropriate test post-hoc for K-W) were marked with appropriate *p*-values. *P*-values < 0.05 were bolded

Bioimpedance spectroscopy revealed a significantly lower lean tissue mass (after adjusting for age and BMI) in the NS, PreD and HD groups in comparison to the control group (*p* = 0.035, *p* = 0.002 and *p* = 0.017, respectively). Similar findings were noted for LTI and BCM. No significant differences in LTM, LTI and BCM were observed between nephrotic patients and participants with advanced deterioration of renal function (PreD and HD group) (Fig. 3a-c).

Severe nephrotic syndrome was found to be associated with a LTM lower by 3.2 kg, corresponding with 3% lower amount of lean tissue (LTM%) when compared to the control group. Similarly, LTI was lower by approximately 0.9 kg/m^2^, and LTI deficit (∆LTI) was more pronounced by about 1.0 kg/m^2^ (Table 2). There was a slightly lower muscle mass in patients with long history of NS in comparison to short history group and MCD diagnosis, but not reaching statistical significance (Additional file Table S1).

**Table 2:** Comparison of BIS parameters of muscle mass in the NS and the control group

BIS parameter of lean tissue		Coefficient with SE	95% CI	***p***-value
**LTM** **adjusted for age**	Control group	*ref.*		
	NS group	−3.17 ± 1.20	−5.59 – −0.77	**0.011**
**LTM%** **adjusted for age**	Control group	*ref.*		
	NS group	−2.97 ± 1.47	−5.93 – −0.02	**0.048**
**BCM** **adjusted for age**	Control group	*ref.*		
	NS group	−2.20 ± 0.05	−3.81 – −0.60	**0.008**
**LTI** **adjusted for age**	Control group	*ref.*		
	NS group	−0.86 ± 0.32	−1.50 – − 0.23	**0.009**
**∆ LTI**	Control group	*ref.*		
	NS group	−0.99 ± 0.33	−1.65 – − 0.33	**0.004**

*SE* standard error, *95% CI* 95% confidence interval of coefficient estimation, *p*-value – significance of comparison of NS and control group in ANCOVA (for LTM, LTM%, BCM and LTI) or ANOVA (for ∆ LTI) test. Statistically significant differences between groups (*p*-values < 0.05) were bolded

We defined a “significant lean tissue deficit” as LTI values below the lower limit of a reference range for age and gender by 1.0 kg/m^2^ (∆LTI ≤ -1.0 kg/m^2^). The significant lean tissue deficit was observed less frequently in the control group (5 patients, 12.5%) than in severe NS (10 patients, 50%; *p* = 0.003) and advanced CKD (PreD+HD groups: 19 patients, 31.7%, *p* = 0.032). No relationship was found between the presence of significant lean tissue deficit and BMI values in the groups (Additional file Table S3).

In patients with different stages of CKD (control, PreD and HD groups taken together) serum albumin concentration positively correlated with lean tissue mass (*r* = 0.35, *p* = 0.032) and index (*r* = 0.31, *p* = 0.059). There was also a moderate inverse correlation between blood urea and both LTM (*r* = − 0.34, *p* = 0.010) and LTI (*r* = − 0.32, *p* = 0.014) in non-haemodialyzed men without NS (control and PreD groups taken together). A strong inverse correlation was observed between blood urea and LTM (*r* = − 0.64, *p* = 0.002), as well as moderate with LTI (*r* = − 0.45, *p* = 0.049) in patients with severe nephrotic syndrome. There was no relationship found between SA and lean tissue descriptors in the NS group.

Adipose tissue mass (adjusted for age) did not differ between the groups (*p* = 0.694) (Fig. 3d). There was a weak correlation between triglycerides and ATM (*R* = 0.34, *p* = 0.003) in CKD patients (control, PreD and HD groups taken together), but no significant relationship was found in the NS group.

Patients with severe nephrotic syndrome were more overhydrated than subjects in the control, PreD and HD groups (Fig. 3e). No significant linear correlations were observed between body water retention (OH) and the degree of proteinuria, hypoalbuminemia or hypoproteinemia in the NS group.

Unadjusted BIS results and statistical significance of covariates are summarized in the additional file (Additional file Table S4-S6).

### Hypercatabolic state assessment

Biochemical nutritional markers and body composition were compared in NS patients, who developed significant hyperphosphatemia (Pi> 5.0 mg/dL) and/or hyperuricemia (UA > 8.0 mg/dL), named hypercatabolic NS, and in those without hPi and hUA called less catabolic NS (Table 3). Younger patient with short NS history and MCD were more likely to be hypercatabolic than those with other underlying glomerulopathies and longer duration of NS. No differences were found between these groups in renal function (serum creatinine, eGFR) and NS severity parameters (serum albumin and total protein concentration, daily proteinuria). Hypercatabolic nephrotic patients had a lower prealbumin concentration and higher serum urea level, as well as lower lean tissue and body cell mass (adjusted for age) than less catabolic subjects. Significant derangements in Pi and/or UA levels were associated with LTM lower by 5.6 kg and BCM by 3.7 kg (after adjusting for age). The hypercatabolic NS group was also found to be more oedematous (*p* = 0.030).

**Table 3:** Characteristic of hypercatabolic and less catabolic patients with severe NS

Variable		Hypercatabolic NS *n = 9*	Less catabolic NS *n = 11*	***p***-value
**History of NS**				
short (< 1 month)	n (%)	7 (77.8%)	3 (27.3%)	**0.035**
long (1–3 months)	n (%)	2 (22.2%)	8 (72.7%)	
**Age** [years]	mean ± SD	37 ± 15	53 ± 16	**0.043**
**Height** [cm]	mean ± SD	176 ± 16	172 ± 13	0.380
**Weight** [kg]	mean ± SD	83 ± 16	83 ± 13	0.910
**BMI** [kg/m^2^]	mean ± SD	27.9 ± 3.3	26.6 ± 3.7	0.423
**eGFR** [mL/min/1.73m^2^]	mean ± SD	85 ± 28	81 ± 31	0.763
**Serum creatinine** [mg/dL]	mean ± SD	1.13 ± 0.3	1.14 ± 0.3	0.982
**Blood urea** [mg/dL]	mean ± SD	70 ± 41	38 ± 13	**0.027**
**Serum albumin** [g/dL]	mean ± SD	1.8 ± 0.4	1.9 ± 0.3	0.538
**Serum total protein** [g/dL]	mean ± SD	4.3 ± 0.4	4.3 ± 0.7	0.703
**Proteinuria** [g/day]	mean ± SD	11.0 ± 6.2	11.9 ± 2.6	0.688
**Prealbumin** [mg/dL]	median (IQR)	18 ± 6	26 ± 8	**0.018**
**OH** [L]	mean ± SD	8.2 ± 5.8	3.8 ± 1.8	**0.030**
**LTM** [kg] adjusted for age	mean ± SD	40.6 ± 2.4	45.8 ± 2.1	0.006*
	B ± SE (95% CI)	−5.6 ± 1.8 (−9.4; −1.9)	*ref*	
**BCM** [kg] adjusted for age	mean ± SD	22.3 ± 1.6	25.6 ± 1.4	0.007*
	B ± SE (95% CI)	−3.7 ± 1.22 (−6.2; − 1.2)	*ref*	

The average values of continuous variables were presented as mean ± SD or median (OQR). Laboratory parameters were compared between groups using independent samples t-test, and BIS parameters (*) using analysis of covariance (ANCOVA) – after an adjustment for age with less catabolic nephrotic patients as a reference group (ref), and described as coefficient (B) with standard error (SE), 95% confidence interval (95% CI). Statistically significant differences between groups (*p*-values < 0.05) were bolded

## Discussion

Renal disorders have been associated with increased risk of malnutrition, particularly along with the progression of CKD. The ISRNM diagnostic criteria have been established to identify patients with PEW accompanying CKD. However, it is not applicable to NS, in which, by definition, the criterion of reduced SA is met, but BMI drop and weight loss are almost never seen, conversely, weight gain due to overhydration is usually observed. Therefore another markers are required to define malnutrition in this group, and to identify hypercatabolic patients, who are especially prone to its development. For this purpose, we have outlined the metabolic pattern of men with severe NS and normal or at most mildly-to-moderately impaired renal in comparison to patients with different CKD stages. To our knowledge, it has been the first clinical investigation that attempts to summarize the clinical expression of a catabolic state in this group of patients.

Body composition derangement was our main area of interest. Although in general population a dual energy X-ray absorptiometry (DEXA) is a gold standard, its value in overhydrated patients is limited. DEXA measurement assess the adipose tissue directly, but the lean tissue is calculated based on ATM without inclusion of hydration status. Thus, in overhydrated individuals, LTM values have been overestimated [11]. Therefore, methods based on bioimpedance techniques, which measure fluid overload aside from body composition, are widely investigated in patients with significant disturbances of hydration status. But BIS also provides results based on equations and assumptions, not direct measurements. Thus, differences between body composition assessed by DEXA and bioimpedance may be observed [12]. However, using the same technique and repeated measurements to follow up the patients status seems to be worth doing.

Over the last decade, various methods based on bioimpedance techniques have been used to assess the nutritional status in patients with CKD, inflammatory bowel disease, neurological diseases or general population from new-borns to elderly [13–17]. However, data regarding the usage of bioelectrical impedance in nephrotic syndrome is limited. So far, bioimpedance technology has been used to estimate the fluid status of patients with nephrotic syndrome rather than body composition [18–21]. We decided to use this method to compare body composition in different groups of patients with kidney disease as simple tool which can be used at the patients’ bedside. Repeated measurements of body composition could add a lot of important information during therapy of nephrotic syndrome.

Based on BIS, our study showed that severe nephrosis was associated with 3.2 kg lower lean tissue mass (3% of LTM), corresponded with an about 1.0 kg/m2 lower LTI in relation to the reference for gender and age (∆LTI) in NS patients when compared to the control group. It made NS group more similar to patients with advanced CKD (PreD+HD) than those with similar renal function (control). The significant lean tissue deficit was noted in the NS group even more often than in PreD and HD patients. The limited number of NS patients, however, made our study underpowered to detect differences in body composition according to the duration of NS.

Interestingly, no agreement between BMI criterion of PEW and the presence of significant LTI deficit was found in any group. It may be explained by the phenomenon of sarcopenic obesity in CKD patients, and by the excess of fluid with an unchanged ATM in the NS group. The significant correlation between triglycerides and ATM in CKD but not in NS, may reflect insulin resistance and support this theory. In the newly diagnosed NS, hypertriglyceridemia has been a result of altered lipoprotein synthesis and metabolism, reflecting the severity of proteinuria rather than insulin resistance. However, the presence of insulin resistance features have been described in longer lasting NS [22].

As the laboratory criteria of PEW have not been reliable for nephrotic patients, we attempted to identify more useful markers of increased catabolism, indicative of the risk of malnutrition in severe NS. We found strong negative correlation between LTM and blood urea concentration, supporting the theory of increased muscle protein degradation in this group [3, 23].

Hyperphosphatemia and hyperuricemia have been other well-known indicators of hypercatabolism. It has been noted in nephrotic children, that hyperphosphatemia (hPi) is attributed to active nephrosis. *Feinstein* et al. suggested that urinary excretion of insulin growth factor 1 (IGF-1) and its influence on increased tubular phosphate retention, may be a possible mechanism of increased phosphorus concentration in this group of children [24]. Thus, hPi has been presumed to be a consequence of hormonal disturbances accompanying massive proteinuria. However, an enhanced catabolism of cellular mass (nucleic acids, adenosine triphosphate – ATP) may be another source of serum phosphate, similarly observed in rhabdomyolysis or tumour lysis syndrome [25, 26]. We suppose that hypercatabolism may be an additional mechanism responsible for hyperphosphatemia in severe NS. Similarly, hPi as an effect of increased catabolism has been described by *Rozentryt* et al. in patients with heart failure [27]. It is probable that the same pathogenetic way is associated with hyperuricemia in nephrotic patients. Hyperuricemic phenomenon has not yet been emphasized in nephrotic syndrome.

In our study, patients with severe NS were divided into two subgroups according to serum concentration of Pi and UA. The subgroup of nephrotic patients with elevated Pi and/or UA levels presented about 5.6 kg lower LTM and 3.7 kg lower BCM with increased blood urea levels and lower serum prealbumin concentration. This may reflect the greater dynamics of muscle tissue catabolism. Considering the fact that patients with a short NS history (and MCD diagnosis) predominated in the hypercatabolic group, the dynamics of NS development may be pivotal.

In our study, patients with severe NS showed a significantly lower lean amount, and features of hypercatabolism (higher Pi, UA and blood urea concentration) in comparison to the control group. In terms of nutritional status, they were more similar to patients with advanced CKD than those with eGFR ≥45 mL/min/1.73m^2^. However, the mechanisms of protein-energy wasting syndrome in CKD (PEW) and NS were significantly different.

In nephrotic patients, lean tissue degradation is a compensatory mechanism that attempts to give a substrate to the synthesis of the lack of plasma protein. However, in severe nephrotic syndrome it is ineffective in elevation plasma protein concentration. The vicious circle phenomenon continues while massive proteinuria persists. In some primary glomerulopathies, such as MN, the inflammation is an underlying cause of the disease and it can enhance the protein degradation [28]. Moreover, proximal tubular cells produce the proinflammatory cytokines in a response to nephrotoxic effect of high protein concentration in the urine on the renal tubules [29]. The inflammatory pathogenesis of PEW in NS appears to be less pronounced than in CKD, but probably not negligible, especially in NS resistant to treatment.

In contrast, in CKD, a chronic non-infectious inflammation, induced in particular by tumor necrosis factor-α and interleukin-6, is the dominant pathway for the development of PEW. Other causes of the wasting are an inadequate calorie intake, reinforced by a diminished appetite due to uremia, multiple comorbid hormonal disorders (insulin and insulin IGF-1 resistance, hypogonadism, hyperparathyroidism) and metabolic acidosis, inhibiting protein synthesis. The disturbances are more strongly expressed along with the progression of CKD [10, 30, 31].

Similar to PEW in CKD, nephrotic syndrome associated PEW is associated with an acceleration in atherosclerosis development, even in children [32]. Not only sarcopenia contributes to it, but also lipid disorders, increased activation of platelets, hypercoagulability, associated hormonal disorders, progressive renal failure [33–37]. Moreover, as we have discovered, hyperphosphatemia and hyperuricemia, recognized independent risk factors of cardiovascular events, were present in patients with severe NS [38–40]. Fluid overload, an independent factor increasing the risk of cardiovascular morbidity and mortality in patients with CKD, was also observed in nephrotic patients [41, 42]. An increased risk of coronary heart disease in nephrotic patients was noted in previous studies [43–45].

As the ISRNM definition of PEW has not been reliable for severe NS, we propose to introduce the term “neph-PEW” to address the characteristic features of nephrotic syndrome associated hypercatabolism and muscle mass loss.

We have identified increased levels of phosphorus, uric acid and blood urea as potential laboratory indicators of enhanced muscle catabolism even in the case of a normal lean tissue amount in BIS examination. We concluded that a decreased lean tissue index compared to the reference value (∆LTI) in BIS examination, in particular a significant lean tissue deficit (∆LTI < -1,0 kg/m^2^), and the presence of severe hyperphosphatemia (hPi: Pi > 5.0 mg/dL) or hyperuricemia (hUA: UA > 8.0 mg/dL) may indicate neph-PEW.

Unfortunately, there are no unequivocal recommendations for dietary management and pharmacological treatment in NS in order to prevent the loss of muscle mass and reduce the risk of cardiovascular complications. Several studies have indicated that a low-protein diet may have a beneficial effect on the reduction of proteinuria and achieving nitrogen balance [1, 5, 46, 47]. However, there is no long-term data regarding the nutritional status of this group of patients. Further longitudinal studies are required to evaluate the process of muscle mass depletion and to assess its dynamics, especially in patients with persistent NS.

Moreover, subsequent studies are required to establish neph-PEW diagnostic criteria and the optimal conservative approach, including nutritional interventions, for reducing the harmful metabolic effects of NS, as well as improving clinical outcomes.

## Conclusions

The results of our study highlighted the problem of hypercatabolic tendencies in severe nephrotic syndrome. Conclusions have been made that a specific metabolic pattern of NS patients may be defined as nephrotic syndrome associated protein-energy wasting (neph-PEW). The study revealed that apart from typical laboratory findings associated with NS such as hypoproteinemia, hypoalbuminemia, hyperlipidemia and hypertrygliceridemia, neph-PEW is expressed as a loss of muscle mass and/or increased levels of biochemical indicators of catabolism – blood urea, phosphorus and uric acid. Bioimpedance spectroscopy may be a helpful tool to identify, among NS patients, those with significant lean tissue deficiencies. The limitation of the study was its relatively small size of study group, as a result of, among other things, the rare occurrence of severe NS. Further research would be required to explore the problem of neph-PEW precisely, and to establish more detailed recommendations for this group of patients.

## Supplementary information

**Additional file 1: Table S1.** Comparison of NS patients with short and long history of nephrotic symptoms before assessment. **Table S2.** Selected PEW criteria of ISRNM evaluated in the groups. **Table S3.** Relationship between PEW criterion of BMI and significant lean tissue deficit in the groups. **Table S4.** Statistical significance of confounding factors for BIS parameters in the groups. **Table S5.** Comparison of unadjusted values of BIS parameters between the groups. **Table S6.** Pairwise comparisons (Bonferroni post-hoc test) for unadjusted values of BIS parameters between the groups.

## Data Availability

The datasets used and/or analysed during the current study are available from the corresponding author on reasonable request.

## Abbreviations

95% CI: 95% confidence interval
ANCOVA: Analysis of covariance
ANOVA: Analysis of variance
ATM: Adipose tissue mass
ATP: Adenosine triphosphate
BCM: Body cell mass
BIS: Bioimpedance spectroscopy
BMI: Body mass index
CKD: Chronic kidney disease
eGFR: Estimated glomerular filtration rate
FSGS: Focal segmental glomerulosclerosis
HD: Haemodialysis
HDL: High-density lipoprotein
hPi: Hyperphosphatemia
hUA: Hyperuricemia
IGF-1: Insulin growth factor-1
ISRNM: International Society of Renal Nutrition and Metabolism
IQR: Interquartile range
K-W: Kruskal-Wallis test
LDL: Low-density lipoprotein
LTI: Lean tissue index
LTM: Lean tissue mass
MCD: Minimal change disease
MDRD: Modification of Diet in Renal Disease
MN: Membranous nephropathy
neph-PEW: Nephrotic syndrome associated protein energy-wasting
NS: Nephrotic syndrome
OH: Overhydration
OR: Odds ratio
PEW: Protein-energy wasting
Pi: Phosphorus
PreD: Predialysis
PSM: Propensity score matching
SA: Serum albumin
SD: Standard deviation
UA: Uric acid
