# Supplementary material for: Protein energy-wasting associated with nephrotic syndrome – the comparison of metabolic pattern in severe nephrosis to different stages of chronic kidney disease
Source: BMC Nephrol. 2020 Aug 14;21:346. doi: 10.1186/s12882-020-02003-4 (PMC7427894; doi:10.1186/s12882-020-02003-4)
Supplement: Supplementary file 1 — Additional file 1: Table S1. Comparison of NS patients with short and long history of nephrotic symptoms before assessment. Table S2. Selected PEW criteria of ISRNM evaluated in the groups. Table S3. Relationship between PEW criterion of BMI and significant lean tissue deficit in the groups. Table S4. Statistical significance of confounding factors for BIS parameters in the groups. Table S5. Comparison of unadjusted values of BIS parameters between the groups. Table S6. Pairwise comparisons (Bonferroni post-hoc test) for unadjusted values of BIS parameters between the groups. [file 12882_2020_2003_MOESM1_ESM.docx]

**Table S1. Comparison of NS patients with short and long history of nephrotic symptoms before assessment.**

| **Variable** | | **Short history of NS**  **(<1 month)**  *n=10* | **Long history of NS**  **(1-3 months)**  *n=10* | **p-value** |
| --- | --- | --- | --- | --- |
| **Glomerulopathy**  MCD  FSGS  MN  AA amyloidosis  AL amyloidosis | n (%)  n (%)  n (%)  n (%)  n (%) | 10 (100%)  0  0  0  0 | 0  1 (10%)  6 (60%)  2 (20%)  1 (10%) | **0.0005** |
| **Age** [years] | mean±SD | 37±13 | 55±17 | **0.017** |
| **Body weight** [kg] | mean±SD | 85±15 | 81±12 | 0.561 |
| **Height** [cm] | mean±SD | 174±11 | 175±9 | 0.913 |
| **BMI** [kg/m^2^] | mean±SD | 27.8±3.9 | 26.5±3.2 | 0.424 |
| **eGFR** [mL/min/1.73m^2^] | mean±SD | 88±24 | 78±34 | 0.465 |
| **Serum creatinine** [mg/dL] | mean±SD | 1.08±0.2 | 1.19±0.4 | 0.421* |
| **Blood urea** [mg/dL] | mean±SD | 58±40 | 48±25 | 0.507 |
| **Serum albumin** [g/dL] | mean±SD | 1.8±0.5 | 1.9±0.4 | 0.549 |
| **Serum total protein** [g/dL] | mean±SD | 4.2±0.5 | 4.4±0.6 | 0.642 |
| **Proteinuria** [g/day] | mean±SD | 11.5±6.0 | 11.5±2.5 | 0.994* |
| **Prealbumin** [mg/dL] | mean±SD | 21.9±16.4 | 23.6±5.3 | 0.650 |
| **Total cholesterol** [mg/dL] | mean±SD | 438±143 | 350±109 | 0.142 |
| **LDL** [mg/dL] | mean±SD | 309±113 | 252±92 | 0.233 |
| **HDL** [mg/dL] | mean±SD | 94±27 | 65±31 | **0.036** |
| **Triglycerides** [mg/dL] | mean±SD | 207±86 | 271±146 | 0.248 |
| **Phosphorus** [mg/dL] | mean±SD | 4.4±0.9 | 4.1±0.7 | 0.421 |
| **Uric acid** [mg/dL] | mean±SD | 8.4±1.2 | 6.8±1.2 | **0.007** |
| **LTM** [kg] | mean±SD | 45.6±10.4 | 41.3±8.4 | 0.322 |
| **LTM adjusted to age** | B ± SE (95% CI) | *ref.* | 0.04±2.34 (-4.90 – 4.98) | 0.986^#^ |
| **LTM%** [%] | mean±SD | 55.1±14.5 | 51.6±11.0 | 0.553 |
| **LTM% adjusted to age** | B ± SE (95% CI) | *ref.* | 0.23±3.36 (-6.86 – 7.31) | 0.947^#^ |
| **BCM** [kg] | mean±SD | 25.6±6.8 | 22.5±5.8 | 0.280 |
| **BCM adjusted to age** | B ± SE (95% CI) | *ref.* | -0.11±1.56 (-3.40 – 3.18) | 0943^#^ |
| **LTI** [kg/m^2^] | mean±SD | 14.9±2.7 | 13.5±2.5 | 0.248 |
| **LTI adjusted to age** | B ± SE (95% CI) | *ref.* | -0.17±0.66 (-0.61 – 0.48) | 0.802^#^ |
| **∆ LTI** [kg/m^2^] | mean±SD | -0.57±2.8 | -1.16±2.6 | 0.632 |
| **ATM** [kg] | B ± SE (95% CI) | 34.2±15.1 | 33.4±10.6 | 0.891 |
| **ATM adjusted to age** | mean±SD | *ref.* | -1.41±3.50 (-8.79 – 5.97) | 0.693^#^ |
| **OH** [L] | median (IQR) | 5.7 (2.7-8.7) | 4.9 (3.6-5.5) | 0.910^ |

*The average values of continuous variables were presented as mean±SD or median (IQR), and were compared between groups with independent samples t-test, Welch test (*), Mann-Whitney test (^). Additionally, ANCOVA was performed (#) with adjustment of BIS results for age with short history group as a reference group (ref), and described as coefficient (B) with standard error (SE) and 95% confidence interval (95%CI). Statistically significant differences between groups (p-values <0.05) were bolded.*

**Table S2. Selected PEW criteria of ISRNM evaluated in the groups.**

| **Criterion** |  | **NS**  *n=20* | **Control**  *n=40* | **PreD**  *n=40* | **HD**  *n=20* | **p-value** |
| --- | --- | --- | --- | --- | --- | --- |
| **SA**  - <3.8 g/dL  - ≥3.8 g/dL | n (%)  n (%) | 20 (100%)  0 | 1 (2.5%)  39 (97.5%) | 4 (10%)  36 (90%) | 4 (20%)  12 (80%) | **<0.0001** |
| **Prealbumin**  - <30 mg/dL  - ≥30 mg/dL | n (%)  n (%) | – | – | – | 2 (10%)  18 (90%) | - |
| **Total cholesterol**  - <100 mg/dL  - ≥100 mg/dL | n (%)  n (%) | 0  20 (100%) | 0  40 (100%) | 0  40 (100%) | 0  20 (100%) | 1.000 |
| **BMI**  - < 23 kg/m^2^  - ≥ 23 kg/m^2^ | n (%)  n (%) | 2 (10%)  18 (90%) | 7 (17.5%)  33 (82.5%) | 3 (7.5%)  37 (92.5%) | 6 (30%)  14 (70%) | 0.117 |
| **PEW diagnosis^#^** | n (%) | **20 (100%)** | **8 (20%)** | **7 (17.5%)** | **9 (45%)** | **<0.0001** |

*^#^At least 1 criterion of PEW according to ISRNM definition. Statistically significant differences between groups (p-values <0.05) were bolded.*

**Table S3. Relationship between PEW criterion of BMI and significant lean tissue deficit in the groups.**

| **BMI values in the groups** | | **∆LTI <-1 kg/m^2^** | **∆LTI ≥-1 kg/m^2^** | **p-value** |
| --- | --- | --- | --- | --- |
| **NS** | **BMI <23 kg/m^2^** | 0 | 2 (20%) | 0.474 |
|  | **BMI ≥23 kg/m^2^** | 10 (100%) | 8 (80%) |  |
| **Control** | **BMI <23 kg/m^2^** | 1 (20%) | 6 (17.1%) | 1.000 |
|  | **BMI ≥23 kg/m^2^** | 4 (80%) | 29 (82.9%) |  |
| **PreD** | **BMI <23 kg/m^2^** | 1 (10%) | 2 (6.7%) | 1.000 |
|  | **BMI ≥23 kg/m^2^** | 9 (90%) | 28 (93.3%) |  |
| **HD** | **BMI <23 kg/m^2^** | 3 (33.3%) | 3 (27.3%) | 0.589 |
|  | **BMI ≥23 kg/m^2^** | 6 (66.7%) | 8 (72.7%) |  |

**Table S4. Statistical significance of confounding factors for BIS parameters in the groups.**

| **BIS parameter** | **Covariates** | |
| --- | --- | --- |
|  | **Variable** | **p-value** |
| **LTM** | Age | **<0.0001** |
|  | BMI | **0.077** |
| **LTI** | Age | **<0.0001** |
| **BCM** | Age | **<0.0001** |
|  | BMI | **0.083** |
| **ATM** | Age | **0.0003** |

*P-values indicating the significance of covariates (p-values <0.1) were bolded.*

**Table S5. Comparison of unadjusted values of BIS parameters between the groups.**

| **Variable** | | **NS**  *n=20* | **Control**  *n=40* | **PreD**  *n=40* | **HD**  *n=20* | **Levene test**  **(p-value)** | **p-value**  **of ANOVA** |
| --- | --- | --- | --- | --- | --- | --- | --- |
| **LTM** [kg] | Mean±SD | 43.4±9.4 | 49.7±9.2 | 42.8±9.4 | 42.9±8.9 | 0.771 | **0.005** |
|  | Min–max | 27.7–58.0 | 32.3–69.2 | 28.1–60.2 | 24.0–57.3 |  |  |
| **LTI** [kg/m^2^] | Mean±SD | 14.2±2.6 | 15.9±2.2 | 14.4±2.9 | 14.0±2.5 | 0.375 | **0.014** |
|  | Min–max | 9.0–18.8 | 12.1–20.9 | 9.0–20.7 | 8.5–17.7 |  |  |
| **∆LTI** [kg/m^2^] | Mean±SD | -0.9–2.7 | 1.1±2.2 | 0.2±2.5 | -0.6±3.1 | 0.421 | **0.022** |
|  | Min–max | -5.6–3.4 | -3.8–6.7 | -4.7–6.2 | -6.6–7.4 |  |  |
| **BCM** [kg] | Mean±SD | 24.1±6.3 | 28.5±6.1 | 23.9±6.5 | 23.7±6.0 | 0.731 | **0.005** |
|  | Min–max | 13.2–33.3 | 17.7–40.7 | 13.3–36.1 | 10.8–33.0 |  |  |
| **ATM** [kg] | Mean±SD | 33.7±12.7 | 36.4±15.9 | 42.5±15.1 | 35.4±19.1 | 0.279 | 0.125 |
|  | Min–max | 9.6–56.4 | 10.2–72.5 | 11.5–71.7 | 10.0–74.8 |  |  |

*SD – standard deviation; min-max: the range from minimum to maximum; Levene test – test of homogeneity of variance, ANOVA – analysis of variance comparing the 4 groups. Statistically significant differences between groups (p-values <0.05) were bolded.*

**Table S6. Pairwise comparisons (Bonferroni post-hoc test) for unadjusted values of BIS parameters between the groups.**

| **Variable** | **Group** | **NS** | **Control** | **PreD** | **HD** |
| --- | --- | --- | --- | --- | --- |
| **ATM** [kg] | **NS** | – | 1.000 | 0.258 | 1.000 |
|  | **Control** | 1.000 | – | 0.448 | 1.000 |
|  | **PreD** | 0.258 | 0.448 | – | 0.590 |
|  | **HD** | 1.000 | 1.000 | 0.590 | – |
| **LTM** [kg] | **NS** | – | 0.095 | 1.000 | 1.000 |
|  | **Control** | 0.095 | – | **0.009** | 0.055 |
|  | **PreD** | 1.000 | **0.009** | – | 1.000 |
|  | **HD** | 1.000 | 0.055 | 1.000 | – |
| **BCM** [kg] | **NS** | – | 0.080 | 1.000 | 1.000 |
|  | **Control** | 0.080 | – | **0.012** | **0.043** |
|  | **PreD** | 1.000 | **0.012** | – | 1.000 |
|  | **HD** | 1.000 | **0.043** | 1.000 | – |
| **LTI** [kg/m^2^] | **NS** | – | 0.110 | 1.000 | 1.000 |
|  | **Control** | 1.110 | – | 0.070 | **0.043** |
|  | **PreD** | 1.000 | 0.070 | – | 1.000 |
|  | **HD** | 1.000 | **0.043** | 1.000 | – |
| **∆LTI** [kg/m^2^] | **NS** | – | **0.040** | 0.735 | 1.000 |
|  | **Control** | **0.040** | – | 0.870 | 0.113 |
|  | **PreD** | 0.735 | 0.870 | – | 1.000 |
|  | **HD** | 1.000 | 0.113 | 1.000 | – |

*Results of Bonferroni post-hoc test were presented as p-values. Statistically significant differences between groups (p-values <0.05) were bolded.*
